# Hepatitis Delta Virus Antigens Trigger Oxidative Stress, Activate Antioxidant Nrf2/ARE Pathway, and Induce Unfolded Protein Response

**DOI:** 10.3390/antiox12040974

**Published:** 2023-04-21

**Authors:** Olga A. Smirnova, Olga N. Ivanova, Furkat Mukhtarov, Vladimir T. Valuev-Elliston, Artemy P. Fedulov, Petr M. Rubtsov, Natalia F. Zakirova, Sergey N. Kochetkov, Birke Bartosch, Alexander V. Ivanov

**Affiliations:** 1 Engelhardt Institute of Molecular Biology, Russian Academy of Sciences, Moscow 119991, Russia; o.smirnova.imb@gmail.com (O.A.S.); olgaum@yandex.ru (O.N.I.); fm277@cornell.edu (F.M.); gansfaust@mail.ru (V.T.V.-E.); fedulovtt@gmail.com (A.P.F.); rubtsov@eimb.ru (P.M.R.); nat_zakirova@mail.ru (N.F.Z.); kochet@eimb.ru (S.N.K.); 2 Centre de Recherche en Cancérologie de Lyon, Université Claude Bernard Lyon 1, INSERM 1052, CNRS 5286, 69434 Lyon, France; birke.bartosch@inserm.fr

**Keywords:** hepatitis delta virus, Nrf2, unfolded protein response, oxidative stress, NADPH oxidase

## Abstract

Hepatitis delta virus (HDV) is a viroid-like satellite that may co-infect individuals together with hepatitis B virus (HBV), as well as cause superinfection by infecting patients with chronic hepatitis B (CHB). Being a defective virus, HDV requires HBV structural proteins for virion production. Although the virus encodes just two forms of its single antigen, it enhances the progression of liver disease to cirrhosis in CHB patients and increases the incidence of hepatocellular carcinoma. HDV pathogenesis so far has been attributed to virus-induced humoral and cellular immune responses, while other factors have been neglected. Here, we evaluated the impact of the virus on the redox status of hepatocytes, as oxidative stress is believed to contribute to the pathogenesis of various viruses, including HBV and hepatitis C virus (HCV). We show that the overexpression of large HDV antigen (L-HDAg) or autonomous replication of the viral genome in cells leads to increased production of reactive oxygen species (ROS). It also leads to the upregulated expression of NADPH oxidases 1 and 4, cytochrome P450 2E1, and ER oxidoreductin 1α, which have previously been shown to mediate oxidative stress induced by HCV. Both HDV antigens also activated the Nrf2/ARE pathway, which controls the expression of a spectrum of antioxidant enzymes. Finally, HDV and its large antigen also induced endoplasmic reticulum (ER) stress and the concomitant unfolded protein response (UPR). In conclusion, HDV may enhance oxidative and ER stress induced by HBV, thus aggravating HBV-associated pathologies, including inflammation, liver fibrosis, and the development of cirrhosis and hepatocellular carcinoma.

## 1. Introduction

Hepatitis delta virus (HDV) is a defective RNA virus that can only propagate in the presence of hepatitis B virus (HBV) [1]. HDV can either infect individuals simultaneously with HBV (co-infection) or patients already bearing HBV (superinfection) [2]. Although co-infection resolves in the majority of cases, superinfection in >90% of cases develops into the chronic stage [3]. Current estimates of chronic hepatitis delta carriers, based on seroprevalence studies, vary from 12 [4] to 62–72 million worldwide [5]. HDV aggravates the course of liver disease by increasing the incidence of acute liver disease during co-infection, and liver fibrosis, cirrhosis, and hepatocellular carcinoma development after superinfection [1,2,3]. On average, HBV/HDV patients develop cirrhosis within 5 years and hepatocellular carcinoma within 10 years [6], highlighting the necessity for investigating the mechanisms of HDV pathogenesis, which are still poorly understood.

HDV virions are comprised of HBV surface antigens and host cell lipids that surround a nucleocapsid formed by a 1679 nt single-stranded circular RNA genome and hepatitis delta antigens [1,7]. After infection with hepatocytes and the translocation of the nucleocapsid into the nucleus, the host cell RNA-polymerase machinery produces three forms of HDV RNA: circular genomic, circular antigenomic, and a linear polyadenylated antigenomic transcript, which acts as messenger RNA [7]. Antigenomic RNA contains a single open reading frame encoding a 195 amino acid protein (p24) [1]. In addition, the circular antigenomic RNA is edited by the host cell’s adenosine deaminase acting on RNA-1 (ADAR-1) at the UAG stop codon, leading to the formation of an amber (W) site [8]. As a result, a second fraction of linear antigenomic RNA is produced in a delayed fashion, bearing an extended ORF that encodes a 214 aa protein (p27). These p24 and p27 proteins are referred to as small (S-HDAg) and large (L-HDAg) HDV antigens [1].

Neither S-HDAg nor L-HDAg exhibit any enzymatic activity. Instead, their role is attributed to the regulation of various stages of the viral life cycle [7]. Much less is known about their role in HDV pathogenesis. To date, it has been shown that they elicit a CD4+ T-cell response, but it does not correlate with the HDV RNA level, i.e., with virus replication, is unknown (reviewed in [9,10]). L-HDAg also induces a CD8+ T-cell response, but, again, its level correlates neither with the HDV RNA levels nor with the incidence of virus resolution. However, several lines of evidence suggest that this response correlates with the severity of liver disease during HDV infection [11,12].

It has also been shown that HDV triggers the production of type I and III interferons (IFN) and concomitant signaling [13], although its antigens also attenuate IFNα signaling by interfering with the JAK/STAT pathway, thus contributing to the establishment of persistent infection and to resistance to treatment with interferon-alpha [14]. L-HDAg potentiates the signaling of a main pro-fibrotic cytokine—transforming growth factor β1 (TGFβ1) [15]—though its effect on the production of TGFβ1 has not been reported so far. The expression of L-HDAg in hepatoma cells has also been shown to promote superoxide anion production accompanied by the induction of NADPH oxidase 4 (NOX4) and activation of NF-κB signaling [16]. However, the authors did not resolve the contraindication between the increased production of superoxide anions and the fact that NOX4 is the only NADPH oxidase that generates hydrogen peroxide [17]. No data exist on the possible interference of HDV with the antioxidant systems of host cells. The authors of the abovementioned paper [16] also presented contradictory data about the induction of the unfolded protein response (UPR) by L-HDAg that is linked to both ROS production and the antioxidant defense Nrf2/ARE pathway [18]. Therefore, the mechanisms of the production and scavenging of reactive oxygen species (ROS) in HDV-infected cells, as well as the influence of viral proteins on the status of the UPR system, require further investigation.

The first goal of our study was to explore the impact of HDV antigens on ROS production and the expression of various ROS-generating enzymes that were previously described for other hepatitis viruses. The second goal was to reveal a possible dysregulation of the antioxidant defense Nrf2/ARE pathway. The third goal was to examine the influence of HDV antigens on ER stress and a consequent UPR. Here, we showed that L-HDAg induces several ROS-producing enzymes, including NOX1 and NOX4, CYP2E1, and Ero1α, activates the Nrf2/ARE defense pathway, and, together with S-HDAg, triggers the UPR.

## 2. Materials and Methods

### 2.1. Reagents

DMEM and Williams E media, FetalClone II, RevertAid reverse transcriptase, Turbofect, and Lipofectamine 3000 and Lipofectamine LTX reagents were from Thermo Fisher Scientific (Waltham, MA, USA); fetal bovine serum (FBS) was purchased from HyClone (Logan, UT, USA); and the other reagents for the cell culture experiments were from Paneco (Moscow, Russia) or Sigma (Taufkirchen, Germany). The Luciferase Assay System kit, Reporter lysis buffer, and pGL3 vectors were supplied by Promega (Madison, WI, USA). The enzymes for cloning were purchased from Fermentas (Vilnius, Lithuania). All other reagents were from Sigma. The High Pure RNA Isolation Kit was from Roche Life Sciences (Basel, Switzerland) and the qPCRmix-HS SYBR mixture was from Evrogen (Moscow, Russia). The oligonucleotides were synthesized by Evrogen. 4-Methylpyrazole (4MP) and diphenyleneiodonium chloride (DPI) were purchased from Sigma. The primary antibodies to heme oxygenase 1 (ab13248), NADPH:quinone oxidoreductase 1 (ab28947), *β*-actin (ab3280), and HRP-conjugated anti-rabbit and anti-mouse secondary antibodies were from Abcam (Cambridge, UK), while the antibodies to NOX1 (Mox1, sc-25545), NOX4 (H-300, sc-30141), Nrf2 (sc-722), and Grp78 (sc-13968) were obtained from Santa-Cruz Biotechnology (Dallas, TX, USA). The antibodies to histone H3 (97155), CHOP (5554), and ATF4 (11815) were provided by Cell Signaling (Leiden, Netherlands). The serum of rabbits immunized with S-HDAg was described earlier [19]. The Huh7 cells were a kind gift from Prof. Ralf Bartenschlager (Heidelberg University, Germany). The pSVLD3 plasmid (#29335) was obtained from Addgene.

### 2.2. Plasmid Construction

The reporter plasmids encoding a firefly luciferase under the control of a promoter with minimal antioxidant response element (ARE, pP-ARE) were described previously [20]. The plasmids pLuc-Nqo1 [21] and pHOGGL3/9.4 [22] with the luciferase gene under the control of Nqo1 or HO-1 gene promoters were a kind gift from Dr. R. Faraonio (Universita` di Napoli Federico II) and Dr. Traylor and Dr. Agarwal (The University of Alabama at Birmingham), respectively. The plasmids with ER stress-inducible elements were constructed based on the pGL3-promoter vector. Briefly, the primers listed in Table 1 were phosphorylated by T4 DNA polynucleotide kinase, annealed, and ligated into pGL3-basic vector linearized by XhoI and KpnI restriction endonucleases. In the case of pP-5xUPRE plasmids, two pairs of primers were used to assemble a sequence bearing five unfolded protein response elements (UPRE). The products were transformed into XL-1 Escherichia coli strain, the clones bearing the target plasmids were selected by the polymerase chain reaction (PCR), and the sequence of the resulting plasmids was verified by Sanger sequencing in the Center of Collective Usage “Genome” (Moscow, Russia).

The plasmids encoding firefly luciferase under the control of Xbp1 or Grp78 promoters were constructed by the amplification of the −273–+222 region of the Grp78 gene promoter or the −340–+107 region of the Xbp1 gene promoter by PCR from genomic DNA of Huh7 cells using the primers listed in Table 1, digesting the purified products with XhoI and KpnI restriction endonucleases, and cloning into the same sites of the pGL3-basic vector.

### 2.3. Cell Culture Experiments and Reporter Assays

Human hepatoma Huh7 cells were maintained in DMEM supplemented with 10% FBS and 2 mM glutamine. HepaRG^NTCP^ cells were cultivated in Williams medium supplemented with 5 µg/mL insulin and 50 µM hydrocortisone, as described in [23]. For transfection, the cells were seeded on 6-well or 24-well plates and transfected at a density of 50–60% with Turbofect and respective plasmids according to the manufacturer’s protocol. Briefly, 2 µg of plasmid and 4 µL of Turbofect per well of a 6-well plate or 0.5 µg of a 4:1 mixture of expression and reporter plasmids and 1 µL of Turbofect per a well of 24-well plate were transfected as previously described [19]. For transfection, mixtures of 1 µg of the plasmid and 2 µL of Lipofectamine 3000 were used per well of a 6-well plate. T-butylhydroquinone (tBHQ) and H_2_O_2_, used as positive controls, were added 20 h post-transfection to 40 and 100 µM, respectively. In the case of reporter plasmids, the cells were lysed 30 h post-transfection by the addition of reporter lysis buffer and incubation at room temperature for 30 min with gentle shaking. The luciferase activity was quantified using the Luciferase Assay System kit according to the manufacturer’s specifications.

For reverse-transcription quantitative PCR (RT-qPCR) or immunoblot analysis, the cells were harvested 48 h post-transfection, if not stated otherwise, and stored at −20 °C.

### 2.4. Quantification of ROS Production

The production of reactive oxygen species was assessed using redox-sensitive fluorescent probes: 2′,7′-dichlorodihydrofluoresceine diacetate (DCFH2DA), dihydroethidium (DHE), and MitoSOX. The cells were transfected on 24-well plates, and 18 h prior to analysis, NOX inhibitor DPI or CYP2E1 inhibitor 4MP were added to 3 µM and 100 µM, respectively. Forty hours after transfection (if not stated otherwise), the medium was removed and a fresh, warm medium containing 25 µM DCFH2DA or DHE or 2 µM MitoSOX was added. After 30 min of incubation at 37 °C, the medium was removed, the cells were washed 10 times with warm phosphate-buffered saline (PBS), and the fluorescence intensity was registered at 485/535 nm (ex/em) in the case of DCFHDA or 510/590 nm (ex/em) in the case of DHE and MitoSOX on a Chameleon V microplate reader (Hydex Oy, Turku, Finland). The levels of fluorescence were normalized to the levels of mock-transfected cells.

### 2.5. Reverse-Transcription Quantitative Polymerase Chain Reaction (RT-qPCR)

Huh7 cells were seeded on 6-well plates, transfected with the respective plasmids as described above, and harvested at 40 h post-transfection if not stated otherwise. RT-qPCR was performed in accordance with [24]. Briefly, the total RNA was isolated with a High Pure RNA Isolation Kit following the standard protocol. Two micrograms of RNA were reverse-transcribed using random hexamer primer and the RevertAid enzyme. The polymerase chain reaction was carried out using the primers listed in Table 2. The standard reaction mixture (10 μL) contained 0.8 µM of forward and reverse primers, cDNA equivalent to 50 ng of total RNA, and a qPCRmix–HS SYBR mixture. The amplification conditions were 55 °C for 5 min, 95 °C for 10 min, followed by 40 cycles each at 95 °C for 10 s, and 57 °C for 1 min (signal collection temperature). The results were analyzed by the ΔΔCt approach.

### 2.6. Western Blotting

Western blot analysis was performed as described previously [25]. Briefly, the proteins were separated in 10% SDS PAGE, transferred to a Hybond ECL membrane (GE Healthcare) that was blocked with 5% (*w/v*) non-fat milk in PBS supplemented with 0.05% (*v/v*) Tween 20 (PBST), incubated with anti-S-HDAg rabbit serum in PBST overnight at 4 °C, and then incubated with HRP-conjugated anti-rabbit antibodies for 1 h at room temperature. The bands were visualized using Pierce ECL Pico Western Blotting Substrate either using ChemiDoc MP equipment (Bio-Rad, Hercules, CA, USA) or on X-ray film.

### 2.7. Infectious HDV Model

HDV virions were obtained by transfection of Huh7 cells at 90% monolayer, cultivated in Williams E medium with 10% FetalClone II, 5 µg/mL insulin, 50 µM hydrocortisone, 50 U/mL penicillin, and 50 µg/mL streptomycin (referred to as “complete Williams medium”, with a mixture of pSVLD3 and pT7HB2.7 plasmids using Lipofectamine LTX according to the standard protocol. Forty-eight hours post-transfection, the medium was replaced with fresh complete Williams medium, and virion-containing conditioned medium was collected six times, starting from 8 days post-transfection, every 48 h. The conditioned medium was combined, and filtered through a 0.22 µm syringe filter, HDV virions were precipitated by the addition of polyethylene glycol 8000 to the final concentration of 8%, gently shaken for 24 h at 4 °C, harvested by centrifugation (3000× *g*, 1 h, 4 °C), and the pellet was resuspended in complete Williams medium and stored in 0.5 mL aliquots at −80 °C;. The titer of virions was quantified by RT-qPCR using the specific primers (Table 2) and expressed as the number of genomic equivalents per milliliter (GE/mL).

HepaRG^NTPC^ cells were seeded in complete Williams medium on 12-well plates at a density of 10^5^ cells/well; after reaching confluency, they were maintained in the same medium without splitting for 5 days, then in the complete Williams medium supplemented with 1.8% DMSO for 3 days, and finally in the same medium with 1 µg/mL tetracycline for 1 day. HDV infection was carried out by the addition of virions at a multiplicity of infection (MOI) of 1000 GE/cell. On the following day, the medium was removed, the cells were washed with phosphate-buffered saline (PBS) (3 × 1 mL per well), and fresh complete Williams medium was added. The infection was monitored by immunostaining using rabbit antibodies to S-HDAg and according to the protocol developed earlier [19].

### 2.8. Statistical Analysis

Statistical analysis was performed with GraphPad Prism 7 (GraphPad Software Inc, Boston, MA, USA). All data are presented as the mean ± standard deviation (S.D.). Differences between two groups were compared by the Welch-corrected unpaired two-tailed Student’s test or by analysis of variance (ANOVA) with Dunett’s post hoc test. *p*-values < 0.05 were considered statistically significant if not stated otherwise.

## 3. Results

### 3.1. Model Description

**Figure 1:**
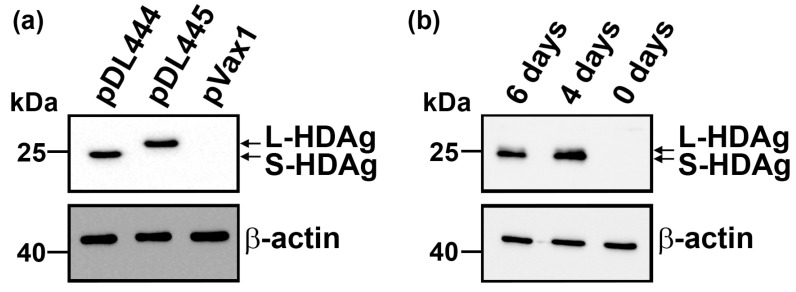
Expression of HDV antigens in non-infectious models. (**a**) Huh7 cells were transfected with plasmids encoding S-HDAg (pDL444), L-HDAg (pDL445), or pVax1 empty vector, and the expression of HDV antigens was analyzed by Western blotting 48 h post-transfection. (**b**) Huh7 cells were transfected with the pSVLD3 plasmid harboring the trimeric HDV genome, and the expression of HDV antigens was assessed by Western blotting 4 and 6 days post-transfection.

The current study is based on two HDV in vitro models: overexpression of individual antigens or their concomitant expression in the context of an autonomously replicating viral genome. Overexpression was achieved by transfecting the plasmids pDL444 and pDL445, which encode S-HDAg and L-HDAg, respectively [26]. Both antigens were readily detected 48 h post-transfection (Figure 1a). Autonomous replication of the HDV genome was established in cells transfected with the plasmid pSVLD3 that encodes three copies of the viral genome. In this case, at least 4 days were required for the detection of productive virus replication (Figure 1b). Huh7 was chosen as a main cell line as it has been widely used in HDV research (for example, [27,28,29]). No effect of HDV expression on the cell proliferation rates or cytotoxicity was observed).

### 3.2. Large Antigen of Hepatitis Delta Virus Triggers Oxidative Stress via Induction of NADPH Oxidases 1 and 4, Cytochrome P450 2E1, and ER Oxidoreductin 1α

The first goal was to evaluate if HDV and its antigens affected the production of reactive oxygen species. As positive controls, treatment with hydrogen peroxide (H_2_O_2_) or tert-butylhydroquinone (tBHQ), chemical inducers of oxidative stress, and transfection with the plasmid encoding HCV core protein were used, as the HCV core is a strong inducer of oxidative stress [25,30]. As a negative control, cells were transfected with the empty pVax1 vector.

**Figure 2:**
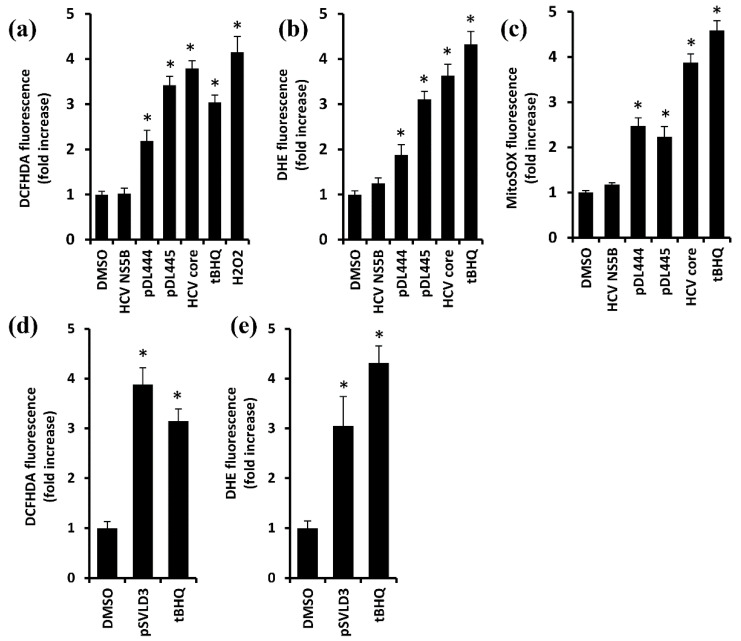
HDV antigens induced the production of reactive oxygen species (ROS). (**a**–**c**) Huh7 cells were transfected with plasmids pDL444 or pDL445 encoding S-HDAg and L-HDAg, or pVax1 as an empty vector; 18 h post-transfection, they were treated with 40 µM tBHQ or 100 µM H_2_O_2_ (where indicated), and 40 h post-transfection, they were stained with ROS-sensitive dyes DCFH2DA (**a**), DHE (**b**), or MitoSOX (**c**). As additional negative and positive controls, the cells were transfected with the plasmids encoding hepatitis C virus NS5B or core proteins. (**d**,**e**) Huh7 cells were transfected with the pSVLD3 plasmid or pVax1 vector (DMSO and tBHQ groups), treated with tBHQ as a positive control or a vehicle carrier DMSO 18 h prior to ROS analysis, and stained with DCFH2DA (**d**) or DHE (**e**) 5 days post-transfection. Bars represent the means ± S.D standardized to empty vector transfection in the presence of DMSO. Statistical significance was analyzed by ANOVA with Dunnett’s post hoc test. * *p* < 0.001.

ROS production was assessed using three redox-sensitive dyes: 2′,7′-dichlorodihydrofluoresceine diacetate (DCFH2DA), dihydroethidium (DHE), and MitoSOX. DCFH2DA are oxidized by hydroxyl radicals and other forms of ROS, as well as by reactive nitrogen species (RNS) [31], so it was used as a probe for the general redox status. DHE and MitoSOX are rather selective probes for superoxide anions in the cytoplasm and mitochondria, respectively [31]. In all these cases, we showed that L-HDAg and, to a lesser extent, S-HDAg increased the rates of ROS production (Figure 2a–c). In the case of MitoSOX, the effect of S-HDAg was statistically insignificant (Figure 2c). Oxidative stress was also detected in cells transfected with pSVLD3 plasmids, i.e., harboring replicating HDV genome (Figure 2d,e).

**Figure 3:**
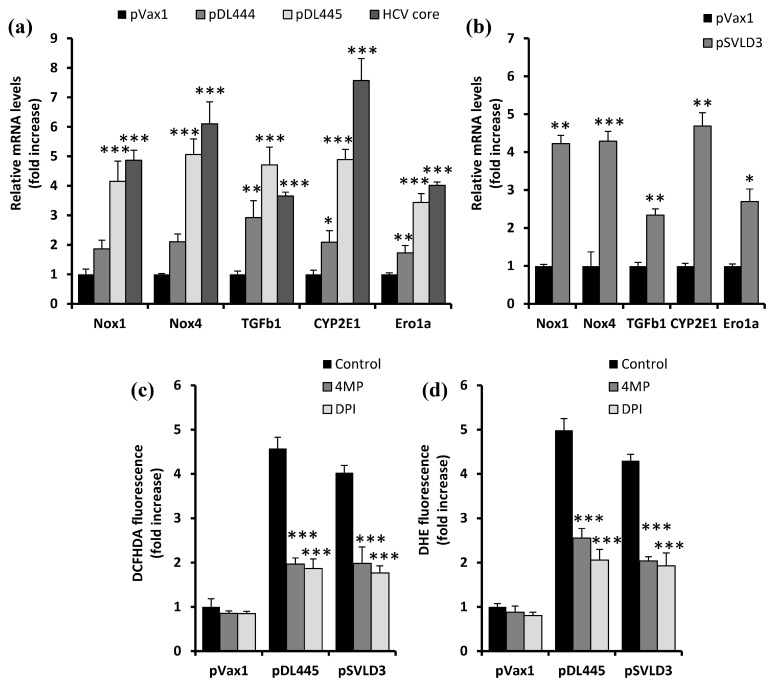
HDV triggers ROS production via the up-regulation of the expression of NADPH-oxidases (Nox) 1 and 4, cytochrome P450 2E1 (CYP2E1), and ER oxidoreductin 1α (Ero1α). (**a**,**b**) Huh7 cells were transfected with plasmids encoding S-HDAg (pDL444), L-HDAg (pDL445), the full HDV genome (pSVLD3), empty pVax1 vector as a negative control, or HCV core protein as a positive control, and 48 h (**a**) or 4 days (**b**) post-transfection, subjected the mRNA levels of ROS-producing enzymes were quantified. As a housekeeping gene, β-glucuronidase (GUS) was used. (**c**,**d**) Huh7 cells were transfected as described above; 18 h prior to analysis, they were treated with 100 µM 4MP or 3 µM DPI, and the ROS production levels were later quantified using DCFHDA (**c**) or DHE (**d**). Bars represent the means ± S.D. Statistical significance was analyzed by ANOVA with Dunnett’s post hoc test (**a**) or an unpaired Student’s test with Welch correction (**b**). (**a**,**b**) * *p* < 0.05, ** *p* < 0.01, *** *p* < 0.001 compared with pVax1-transfected cells, (**c**,**d**) *** *p* < 0.001 vs. DMSO-treated cells transfected with the respective plasmid.

As ROS are produced by a variety of cellular enzymes and systems, we evaluated the impact of HDV on the expression of extramitochondrial ROS-producing enzymes that were previously described to mediate the induction of oxidative stress by HCV infection. These included NADPH oxidases 1 and 4 (NOX1, NOX4), cytochrome P450 2E1 (CYP2E1), and ER oxidoreductin 1α (Ero1α). As shown in Figure 3, both the overexpression of HDV antigens and replication of its RNA in Huh7 cells led to a significant increase in the mRNA levels of these enzymes. Again, the effect of S-HDAg was much less pronounced than that of L-HDAg. These results suggest that HDV triggers oxidative stress by the induction of NADPH oxidases 1 and 3, liver-specific cytochrome CYP2E1, and a component of protein folding machinery of the ER—Ero1α. To ensure that these enzymes were indeed responsible for HDV-induced increased ROS production, in a separate experiment, the pDL445- and pSVLD3-transfected cells were treated with diphenyleneiodonium chloride (DPI), which inhibits NADPH oxidases [32] or 4-methylpyrazole (4MP), a CYP2E1 inhibitor [33]. Both compounds decreased the levels of ROS production, as revealed in both the DCFH2DA and DHE assays (Figure 3c,d), although not to the level of cells transfected with the empty vector.

### 3.3. Expression of the Large HDV Antigen Activates Antioxidant Defense Nrf2/ARE Pathway

**Figure 4:**
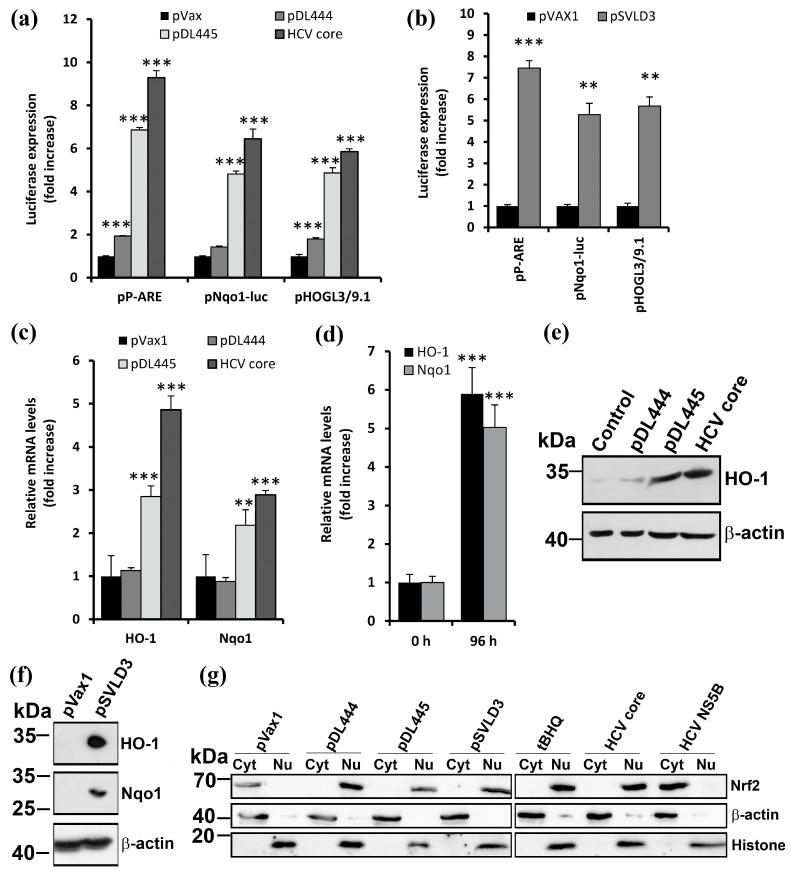
HDV antigens activate the antioxidant Nrf2/ARE pathway. (**a**,**b**) Huh7 cells were co-transfected with plasmids encoding S-HDAg (pDL444), L-HDAg (pDL445), the full HDV genome (pSVLD3), empty pVax1 vector as a negative control or HCV core protein as a positive control with the reporter plasmids encoding a luciferase gene under the control of the SV40 promoter with minimum ARE (pP-ARE) or promoters of Nqo1 (pNqo1-luc) or HO-1 (pHOGL3/9.1). Two (**a**) or four (**b**) days post-transfection, luciferase expression was quantified by assaying its enzymatic activity in cell lysates. (**c**–**g**) Huh7 cells were transfected with the above-mentioned plasmids, and the mRNA (**c**,**d**) or protein (**e**,**f**) levels were analyzed by RT-qPCR and Western blotting, respectively. (**g**) The cytoplasmic (Cyt) and nuclear (Nu) protein fractions were separated using an NE-PER kit, and the levels of Nrf2, as well as β-actin and histone H3 as control cytoplasmic and nuclear proteins, were assessed in them by western blotting. Bars represent the means ± S.D. Statistical significance was analyzed by ANOVA with Dunnett’s post hoc test (**a**) or an unpaired Student’s test with Welch correction (**b**). ** *p* < 0.01, *** *p* < 0.001 vs. to pVax1-transfected cells.

The status of the Nrf2/ARE pathway in Huh7 cells expressing HDV antigens was evaluated using several approaches, including the use of reporter plasmids, analysis of Nrf2 intracellular localization, and quantification of the relative expression of Nrf2-dependent genes. The co-transfection of Huh7 cells with HDV antigen-expressing plasmids and a reporter plasmid encoding a luciferase under the control of SV40 promoter with the introduced ARE sequence [20] revealed that L-HDAg induced a fivefold increase in luciferase expression (Figure 4a). In contrast, luciferase expression in cells bearing S-HDAg was comparable to that in cells transfected with the empty vector. A similar result was obtained using the reporter plasmids, in which luciferase expression was controlled by large ARE-containing regions of NADPH:quinone oxidoreductase 1 (Nqo1) or heme oxygenase 1 (HO1) promoters. L-HDAg, but not S-HDAg, also potentiated the expression of endogenous Nqo1 and HO1, as was shown by both RT-qPCR and Western blot analysis (Figure 4c,e). To verify the activation of the Nrf2/ARE pathway, we assessed the intracellular localization of Nrf2 by the transfection of Huh7 cells with HDV antigen-encoding plasmids, separation of nuclear and cytoplasmic fractions using a commercial NE-PER kit, and detection of Nrf2 in these fractions using standard Western blotting. To control the separation of nuclear and cytoplasmic fractions, histone H3 and β-actin were detected. In the cells transfected with the empty vector or the plasmid for the expression of the HCV NS5B protein that does not activate the Nrf2/ARE pathway [20], Nrf2 localized to the cytoplasm (Figure 4g). In the cells expressing S-HDAg or L-HDAg, or the control HCV core protein, or those treated with tBHQ, Nrf2 was found predominantly in the nucleus. Similar changes were observed in the Huh7 cells transfected with the pSVLD3 plasmid, i.e., expressing the virus antigens in the context of its RNA. Therefore, the HDV large antigen activated the antioxidant Nrf2/ARE pathway (Figure 4b,d,f,g).

### 3.4. Large HDV Antigen Provokes ER Stress and Concomitant Unfolded Protein Response

The next goal was to determine if HDV triggered ER stress and unfolded protein responses. This involved the activation of three key mediators: transcription factor ATF6, endonuclease Ire1, and a PKR-like ER-residing protein kinase (PERK). UPR induction led to increased expression of ER chaperones and other proteins involved in protein folding, the activation of the ER-associated protein degradation (ERAD) system, block of cap-dependent translation via the phosphorylation of eIF2α factor, and the induction of proapoptotic proteins ATF4/CREB2 and CHOP/GADD153. 

**Figure 5:**
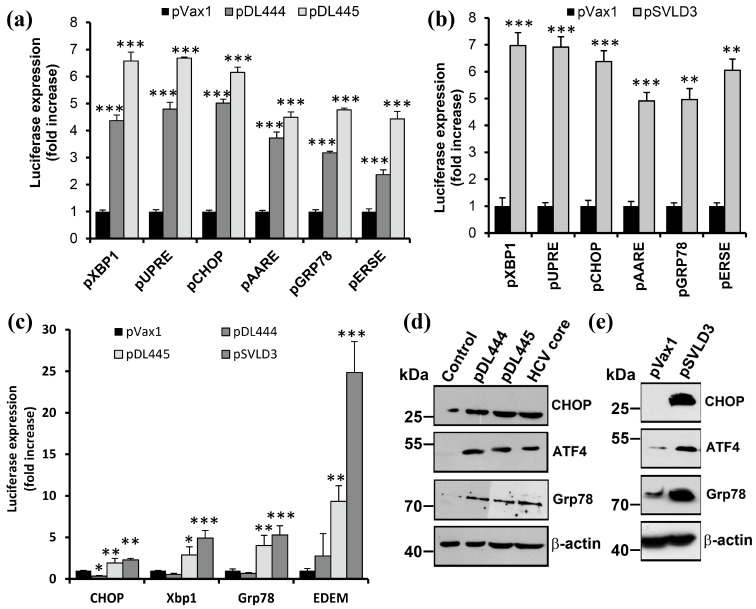
Large HDV antigen induced unfolded protein response. (**a**,**b**) Huh7 cells were co-transfected with plasmids encoding S-HDAg (pDL444), L-HDAg (pDL445), the full HDV genome (pSVLD3), or empty pVax1 vector as a negative control with the reporter plasmids encoding a luciferase gene under the control of the SV40 promoter with minimum UPRE (pUPRE), ERSE (pERSE), or AARE (pAARE), or promoters of Xbp1 (pXbp1), CHOP (pCHOP), or Grp78 (pGRP78). Two (**a**) or four (**b**) days post-transfection, luciferase expression was quantified by assaying its enzymatic activity in cell lysates. (**c**–**e**) Huh7 cells were transfected with the above-mentioned plasmids, and the mRNA (**c**) or protein (**d**,**e**) levels were analyzed by RT-qPCR and Western blotting, respectively. The β-actin blot in panel (**e**) was also used in panel 4f, as they came from the same experiment. Bars represent the means ± S.D. Statistical significance was analyzed by ANOVA with Dunnett’s post hoc test (**a**) or unpaired Student’s test with Welch correction (**b**). * *p* < 0.05, ** *p* < 0.01, *** *p* < 0.001 vs. pVax1-transfected cells.

The analysis was performed following several approaches. The first was the usage of reporter plasmids encoding a luciferase under the control of UPR-inducible genes or their consensus response elements: UPRE, ER stress response element (ERSE), or amino acid response element (AARE). The other approaches were standard RT-qPCR and immunoblotting. The results are presented in Figure 5. It can be clearly seen that the overexpression of either HDV antigen or the replication of the viral RNA pronouncedly increased the activity of UPRE-, ERSE-, or AARE-containing promoters (Figure 5a,b), enhanced the transcription of UPR-inducible genes (Figure 5c), and led to their accumulation (Figure 5d,e). It is noteworthy that S-HDAg also caused a strong and statistically significant effect.

### 3.5. Current Infectious Models Do Not Allow Analysis of Changes Specifically in Infected Cells

**Figure 6:**
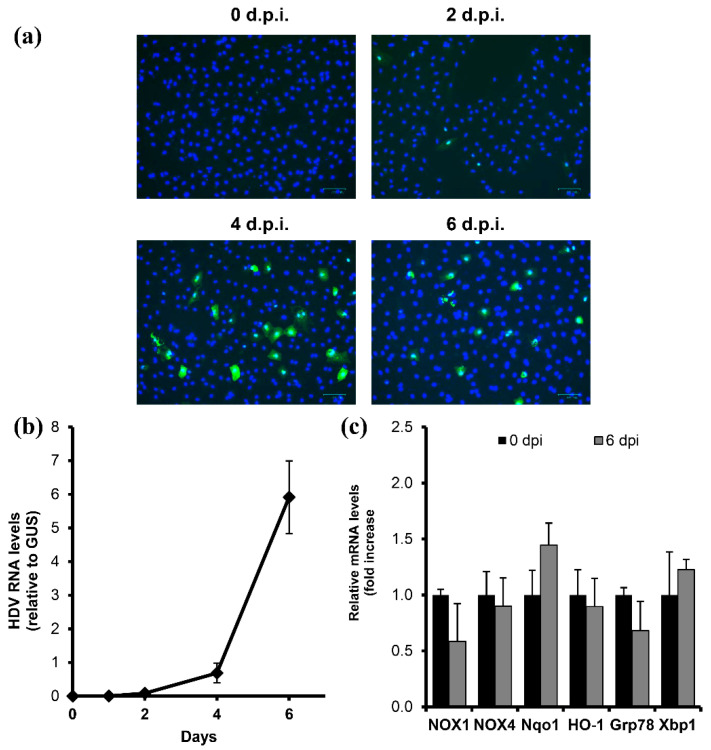
Infectious HDV model did not allow monitoring of virus-induced changes in redox pathways. HepaRGNTCP cells were infected with HDV, and the levels of infection were analyzed by immunostaining (**a**) or RT-qPCR (**b**) until 6 days post-infection (d.p.i.). (**c**) Transcription of ROS-generating and Nrf2-enzymes, as well as of UPR-dependent genes, was assessed by RT-qPCR. Graphs represent the means ± S.D.

Finally, we aimed to verify the effects described above in infectious models, specifically in the HepaRG cell line overexpressing the NTCP receptor (HepaRG^NTPC^). The cells kept in a monolayer for 8 days and treated with tetracycline to induce the receptor were infected with HDV at an MOI of 40 GE/cell, as described previously. HDV RNA was detected starting from 48 h post-infection and reached maximum levels to day 6 (Figure 6a,b), at which point the analysis was carried out. The expression of ROS-generating enzymes and Nrf2-dependent and UPR-induced genes was assessed by RT-qPCR. However, no induction was observed for either of them (Figure 6c). As the negative effect could be due to the low percentage of infected cells, the latter was monitored by immunofluorescence. It revealed that no more than 10% of cells were infected. Therefore, this, and potentially other infectious HDV models, are inapplicable for the analysis of events occurring in infected cells only.

## 4. Discussion

Oxidative stress, i.e., the overproduction of reactive oxygen species to the levels at which they cannot be efficiently neutralized, can be hazardous to biomolecules, including DNA, proteins, and lipids [34]. Moreover, as ROS act as signaling molecules, their excessive production can affect a variety of cellular signaling pathways, leading to the modulation of cell growth and differentiation [35,36]. Various viruses have been shown to trigger massive ROS production [37,38,39,40,41]. Specifically, pronounced oxidative stress occurs during chronic hepatitis B and C infections [42]. In these cases, oxidative stress has been linked to the development of inflammation, fibrosis, and survival of infected cells, with all of these factors leading to liver cirrhosis and cancer [43]. Our results suggest that, upon co-infection, HDV may further augment ROS production. Therefore, HDV-induced oxidative stress could be another driving factor of viral pathogenesis.

Viruses can induce oxidative stress via various mechanisms: by inducing mitochondrial dysfunction via altered calcium homeostasis or the induction of NADPH-oxidases, catabolic enzymes (xanthine oxidase, spermine oxidase), CYP2E1, or Ero1α [39,40,42,44]. Previously, we demonstrated that HCV proteins trigger ROS production via the induction of NOX1, NOX4, CYP2E1, or Ero1α [25,30,44,45]. In the present paper, we show that the same mechanisms modulate the response to HDV replication. This suggests that these two hepatitis viruses may employ similar mechanisms of oxidative stress induction. The involvement of NOX4, a membrane protein found on the nuclear membrane [44], may indicate that H_2_O_2_ is produced in close proximity to DNA, thus leading to its damage [44]. The upregulation of CYP2E1 by HDV infection suggests that this virus can aggravate alcoholic liver disease (ALD), as heavy alcohol consumption is accompanied by an increase in ER mass and the expression of the ER-residing CYP2E1 [46].

One of the major challenges in studies of virus-induced oxidative stress is the lack of experimental data showing sites within the infected cell where ROS production is enhanced. Most groups, including ours, study ROS production using redox-sensitive fluorescent dyes (DCFH2DA, DHE, and MitoSOX). In addition to their specificity issue, they do not clarify whether ROS are produced at a specific site in a cell. Even the mitochondrial matrix-targeted MitoSOX dye that reacts with superoxide anions does not exclude reactions with ROS that migrated from the intermembrane space or cytoplasm. However, it is worth noting that biological membranes, with the exception of the mitochondrial outer membrane, are considered relatively impermeable to superoxide anions [47,48]. Therefore, future endeavors can involve the analysis of ROS gradients using more specific probes, such as HyPER proteins targeted to various organelles (nucleus, mitochondrial matrix, or intermembrane space) or its conjugates with tubulin/actin to prevent the diffusion of the probe. This approach has been successfully implemented by Belousov’s group in non-infectious models [49]. Alternatively, sites of ROS production can be identified by the detection of hyperoxidized forms of peroxiredoxins—the most potent peroxide scavengers [35,50].

Scavenging of ROS is achieved by two families of enzymes, namely peroxiredoxins (Prdx) and glutathione peroxidases (GPx), which have high affinity to hydrogen peroxide [50,51]. The oxidized forms of these enzymes are recycled by thioredoxin and glutaredoxin systems that rely on antioxidant glutathione and the enzymes of its biosynthesis and regeneration, as well as on thioredoxin and thioredoxin reductase. The expression of many of these enzymes, as well as some forms of Prdx, is controlled by a redox-sensitive transcription factor, Nrf2 [52]. Nrf2 recognizes a conservative antioxidant response element (ARE) in its promoters [52]. Numerous data exist in the literature showing that the activation or suppression of the Nrf2/ARE pathway is strongly associated with various pathologies, including carcinogenesis [53] and inflammatory diseases [54]. During the last decade, several groups, including ours, reported that hepatitis B and C viruses, as well as other oxidative stress-inducing pathogens, dysregulate the Nrf2/ARE system [20,55,56,57]. Here, we show that HDV antigens activate the Nrf2 factor. As the hepatitis B virus was previously reported to activate Nrf2 signaling both in vitro and in the liver [56], it is tempting to speculate that HDV can further enhance signaling. In addition, the Nrf2 factor plays a significant role in the regulation of cell metabolism, as it controls the expression of several key glycolytic genes. Therefore, the activation of the pathway may also rewire cell metabolism in infected cells, which merits further studies.

The cellular redox status is also interconnected with endoplasmic reticulum (ER) stress and the consequent unfolded protein response (UPR) [58]. The ER is an organelle responsible for the folding of most proteins, as well as for the biosynthesis of sterols and lipids. Chemical or biological agents that alter various processes in the ER and trigger the accumulation of misfolded proteins activate a special program referred to as UPR [58]. Persistent UPR may result in cell death via apoptosis, necroptosis, or autophagy [58]. It has been linked to the development of a wide spectrum of pathologies, including alcoholic- and non-alcoholic liver disease, various autoimmune and inflammatory disorders, and neurodegenerative diseases [59]. Several viruses have been shown to induce UPR, including SARS-CoV-2, HCV, and HBV [42,60]. The above-cited paper of Williams et al. states that HDV antigens do not induce UPR, despite their data on the increased expression of a reporter under the control of an ERSE element that is one of the three key elements of UPR pathways [16]. However, our data clearly show that HDV replication induces ER stress and, concomitantly, UPR.

UPR is often confused with another event that originates from the ER–ER overload response (EOR) [61]. EOR occurs during the excessive/massive synthesis and folding of proteins in this organelle without the accumulation of misfolded proteins. In contrast to UPR, UOR directly triggers significant ROS production via the efflux of calcium ions from the ER [61]. It is also accompanied by the activation of the redox-sensitive STAT3 and NFκB transcription factors. HCV proteins induce not only UPR, but also EOR [62,63]. Therefore, the previously published data of Williams et al. [16] suggest that L-HDAg induces ER overload response, while our findings imply that the antigen also triggers UPR. It is possible that HDV augments ER stress caused by HBV and its HBx, surface (HBcAg), or envelope (HBeAg) proteins.

One of the major limitations of this study is the absence of data from infectious cell models. Current in vitro systems that reproduce all stages of the virus life cycle are based on HepaRG, HepaRG^NTCP^, or HepG2^NTPC^ cell lines that express sodium/taurocholate cotransporting polypeptide (NTCP, SLC10A1), a putative receptor for HDV and HBV [64,65]. However, even its overexpression in any of these cell lines does not allow the infection of the majority of cells in a culture. The typical infection rates do not exceed 10% [64,65]. Therefore, the changes in the expression of the genes in virus-infected cells cannot be registered if these genes are expressed in uninfected cells. The exception is genes, such as cytokines, that are not expressed in the absence of infection [13]. The enhancement of infection efficiency by the discovery of other, undiscovered, factors of cell permissiveness to HDV or the development of approaches for the selection of virus-infected cells without disrupting the cell phenotype/differentiation status may allow the infected cells to be examined, instead of analyzing a mixture of infected and uninfected cells.

Another limitation of this study is the choice of the Huh7 cell line for HDV replication and the overexpression of its antigens. This cell line was widely used in the field, not only before the discovery of HepaRG, but also in recent years (for example, see [28]). Since then, these hepatoma cells were substituted either with another hepatocellular carcinoma HepG2^NTPC^ cell line or with liver progenitor HepaRG cells that do not display the features of tumor cells. However, HepaRG cells require a four-week differentiation procedure, which limits their experimental usefulness [23]. In contrast, the HepaRG^NTCP^ cell line allows infection right after a short differentiation protocol [64]. Here, we verified the key findings using this cell line.

## 5. Conclusions

Our data provide experimental evidence that HDV replication in liver cell lines and the concomitant expression of the viral antigen(s) trigger ROS production through the overexpression of a series of ROS-generating extramitochondrial enzymes, activates the Nrf2/ARE pathway that controls the expression of antioxidant enzymes, and induces ER stress and concomitant unfolded protein response. Future studies can show the significance of these events in HDV pathology.

## Figures and Tables

**Table 1:** Primers used for plasmid construction.

Plasmid	Orientation ^1^	Sequence (5′-3′)	
pGL3-5xUPRE	Dir	CACAGGTGCTGACGTGGCATTCACAGGTGCTGACGTGGCATTCACAGGT and GCTGACGTGGCATTCACAGGTGCTGACGTGGCATTCACAGGTGCTGACGTGGCATTC	
	Rev	GTGAATGCCACGTCAGCACCTGTGAATGCCACGTCAGCACCTGTGGTAC and TCGAGAATGCCACGTCAGCACCTGTGAATGCCACGTCAGCACCTGTGAATGCCACGTCAGCACCT	
pGL3-ERSE	Dir	CCACCAATCGGAGGCCTCCACGACCACCAATCGGAGGCCTCCACGAC	
	Rev	TCGAGTCGTGGAGGCCTCCGATTGGTGGTCGTGGAGGCCTCCGATTGGTGGGTAC	
pGL3-AARE	Dir	CAACATTGCATCATCCCCGCAACATTGCATCATCCCCGCC	
	Rev	TCGAGGCGGGGATGATGCAATGTTGCGGGGATGATGCAATGTTGGTAC	
pGL3-Xbp1	Dir	TTCCCTCGAGCGACAGAAGCAGAACTTTAG	
	Rev	TTCCGGTACCCCTGAGGTAATTCTCTGTTAG	Gene ID: 7494
pGL3-Grp78	Dir	TTCCCTCGAGCTTCATCTTGCCAGCCAGT	Gene ID: 3309
	Rev	TTCCGGTACCCGAGATAGACAGCTGCTGAACCA	

^1^ Dir—direct, Rev—reverse.

**Table 2:** Primers used for RT-qPCR analysis.

Gene	Sequence (5′-3′)	GenBank Accession No.
Nox1	CAATCTCTCTCCTGGAATGGCATCCT	NM_007052.5
	CCTGCTGCTCGGATATGAATGGAGAA	
Nox4	CTGCATGGTGGTGGTGCTAT	NM_016931.5
	CCGGGAGGGTGGGTATCTAA	
TGFβ1	TGGCGATACCTCAGCAAC	NM_000660.7
	ACCCGTTGATGTCCACTTG	
COX2	GCCAAGCACTTTTGGTGGAG	NM_000963.4
	GGGACAGCCCTTCACGTTAT	
CYP2E1	TTTAAGCCAGAACACTTCC	NM_000773.4
	GCACACAACAAAAGAAACA	
Ero1α	TCATTGAAGAATGTGAACAA	NM_001382464.1
	ATCATGCTTGGTCCACTGAA	
Nqo1	CCGTGGATCCCTTGCAGAGA	NM_000903.3
	AGGACCCTTCCGGAGTAAGA	
HO-1	CCAGCAACAAAGTGCAAGATTC	NM_002133.3
	TCACATGGCATAAAGCCCTACAG	
CHOP	AGAACCAGGAAACGGAAACAGA	NM_001195053.1
	TCTCCTTCATGCGCTGCTTT	
Xbp1	GTGCAGGCCCAGTTGTCACC	NM_005080.4
	TCTGGGTAGACCTCTGGGAG	
Grp78	CCACCTCCAATATCAACTTG	NM_005347.5
	ACGATCAGGGCAACCGCATCA	
EDEM	CTGGGTTGGAAAGCAGAGTG	NM_014674.3
	TCTCCTTCATTGCAGGCTTC	
GUS	CGTGGTTGGAGAGCTCATTTGGAA	NM_000181.4
	ATTCCCCAGCACTCTCGTCGGT	
HDV	GGACCCCTTCAGCGAACA	M21012.1
	CCTAGCATCTCCTCCTATCGCTAT	

## Data Availability

The data presented in this study are available on request from the corresponding author.
